# Predictive Value of QRS Fraction for Cardiovascular Death in Patients with Heart Failure: A Prospective Cohort Study in Acute Decompensated Heart Failure (Heb-ADHF)

**DOI:** 10.31083/j.rcm2307241

**Published:** 2022-06-27

**Authors:** Xiaoran Cui, Demin Liu, Xue Geng, Qian Wang, Ruibin Li, Wenli Zhou, Wei Cui

**Affiliations:** ^1^Department of Cardiology, The Second Hospital of Hebei Medical University, 050004 Shijiazhuang, Hebei, China

**Keywords:** heart failure, QRS fraction, HFpEF, cardiovascular death, prognosis

## Abstract

**Background:**

The QRS fraction is the ratio of the total amplitude of R waves to the total amplitude of QRS complexes (∑R/QRS) on a 12-lead electrocardiogram. Our group has previously proposed calculation of the QRS fraction as a simple method for estimation of left ventricular ejection fraction. In this study, we explored the ability of the QRS fraction to predict cardiovascular death in patients with heart failure.

**Methods:**

The study had a prospective, observational design and collected epidemiological and follow-up data for 1715 patients with heart failure who were inpatients in the Department of Cardiology at the Second Hospital of Hebei Medical University between January 2017 and December 2018. The patients were stratified according to quartile of QRS fraction, namely, lower (<43.8%, Q1 group) middle (43.8%–61.0%, Q2 group), and higher (>61.0%, Q3 group).

**Results:**

One thousand and fifty-one (61.28%) of the 1715 patients were male and the median follow-up duration was 261 days (interquartile range 39, 502). There were 341 (19.88%) deaths, including 282 (16.44%) with a cardiovascular cause. The Q1, Q2, and Q3 groups comprised 431 (25.13%), 850 (49.56%), and 434 (25.31%) patients, respectively. There were significant differences in cardiovascular mortality among the three QRS fraction subgroups (*p *< 0.05). Kaplan-Meier survival curves of different QRS fraction levels showed significant diffference among patients with heart failure, especially among those with preserved ejection fraction (*p* = 0.025 and 0.031, log-rank test). Cox regression analysis showed that the QRS fraction was independently associated with the risk of cardiovascular death. The risk of cardiovascular death was lower in the Q2 and Q3 groups than in the Q1 group, with respective hazard ratios of 0.668 (95% confidence interval 0.457–0.974) and 0.538 (95% confidence interval 0.341–0.849).

**Conclusions:**

The QRS fraction may serve as a prognostic indicator of the long-term risk of cardiovascular death in patients with heart failure, especially those with preserved ejection fraction.

**Clinical Trial Registry:**

ChiCTR-POC-17014020.

## 1. Introduction

Heart failure is a growing public health problem that is an important cause of death and disability globally and a source of increasing health care costs year after year [1, 2, 3]. Current reports estimate that more than 26 million people worldwide have the burden of heart failure [4, 5, 6]. Studies in several countries have shown that survival in patients with heart failure improved markedly between 1980 and 2000 [7, 8, 9, 10, 11]. However, since then, this positive trend has levelled off [12]. In the ESC Heart Failure Long-Term Registry (ESC-HF-LT) [3], the rate of all-cause mortality at 1 year has been reported to be 8.1% (including heart failure with reduced ejection fraction [HFrEF], heart failure with mid-range ejection fraction [HFmrEF], and heart failure with preserved ejection fraction [HFpEF]), and 52.1% of deaths have been attributed to cardiovascular causes [3]. In the Italian Heart Failure Registry (IN-HF, Italian Network on Heart Failure) [13], the cumulative total mortality rate at 1 year has been reported to be 24% in acute heart failure (19.2% in 797 patients with heart failure de novo and 27.7% in 1058 with worsening heart failure) and 5.9% in chronic heart failure (CHF). Therefore, cardiovascular deaths account for 73.1% and 65.3% of total mortality in acute heart failure and chronic heart failure, respectively.

Changes in cardiac structure and function often precede clinical manifestations, and certain alterations are shown on the electrocardiogram (ECG) [14, 15]. The standard 12-lead ECG is a low-cost, convenient, rapid, and widely used method. Changes in electrical activity cause changes in mechanical activity, resulting in changes in hemodynamics, with the result being a decrease in cardiac pumping function and an increased risk of death. Several ECG markers, including QRS duration, fragmented QRS (fQRS), and the QRS-T angle, have been identified to have the potential to predict the risk of adverse events [16, 17]. QRS prolongation (>120 ms) can reflect left ventricular systolic dysfunction. In patients with HFrEF, QRS prolongation is thought to be associated with increased mortality and sudden death in patients with heart failure [17, 18, 19]. Therefore, patients with HFrEF and a QRS duration ≥130 ms can be considered for cardiac resynchronization therapy with a defibrillator. The presence of an fQRS on ECG in patients with heart failure suggests the presence of ventricular asynchrony [20]. The previous study shows that fQRS is an independent predictor of cardiovascular mortality, ventricular arrhythmias, and sudden cardiac death in Asian patients with heart failure in association with ischemic cardiomyopathy. However, fQRS does not have predictive value for cardiovascular death in patients with non-ischemic cardiomyopathy [21]. The spatial QRS-T angle is the angle between the direction of ventricular depolarization and that of repolarization. Previous studies have shown that the frontal QRS-T angle is a predictor of increased morbidity and mortality in patients with CHF [22] and in patients with diastolic heart failure [23]. However, widened QRS-T angles are commonly associated with left ventricular hypertrophy (LVH), bundle branch block, pacing, and ischemia.

Based on previous studies, our group proposed a simple method for estimation of left ventricular ejection fraction (LVEF) whereby the ratio of the total amplitude of R waves to the total amplitude of QRS complexes (∑R/∑QRS), known as the QRS fraction, is calculated on the 12-lead ECG [24, 25] and can be used in patients with and without myocardial infarction [26]. The QRS fraction is simple to calculate and, unlike the above-mentioned ECG indicators, is not affected by left ventricular hypertrophy and other factors. However, in the past, the QRS fraction has not been used for prognostic assessment. The aim of this study was to investigate the ability of the QRS fraction to predict the long-term prognosis in patients with heart failure and to explore its role in predicting cardiovascular death.

## 2. Methods

### 2.1 Study Design and Participants

**Fig. 1.**
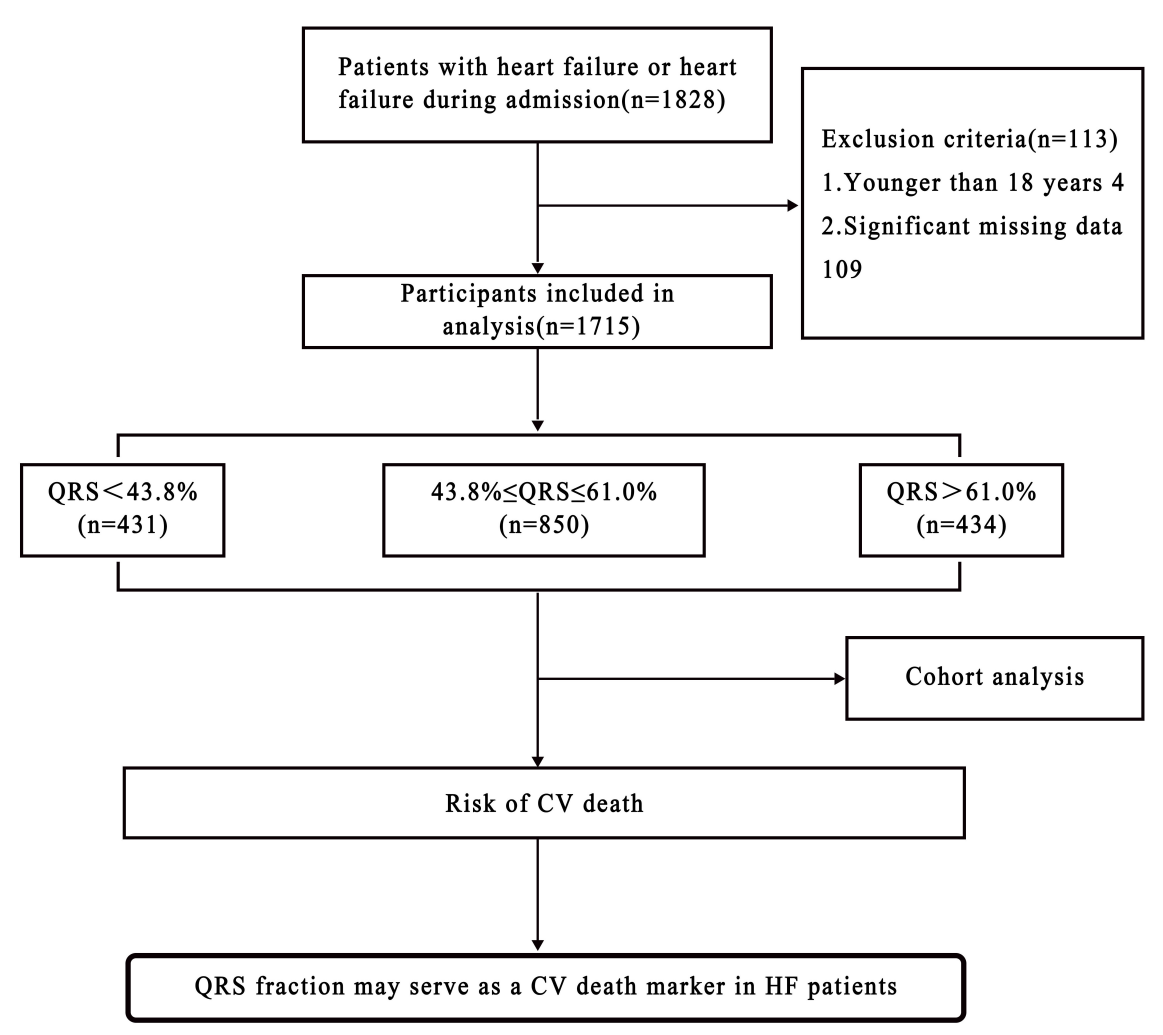
**Flow-diagram**.

The study participants were 1715 consecutive patients (male, 61.28%; female, 38.72%) with heart failure who were hospitalized in the Department of Cardiology at the Second Hospital of Hebei Medical University between January 2017 and December 2018. Heart failure was diagnosed by at least two experienced cardiologists and in accordance with the 2016 European Society of Cardiology guidelines for the diagnosis and management of acute heart failure and CHF [27, 28]. The study inclusion criteria were as follows: aged ≥18 years; clinical symptoms or signs of heart failure at the time of admission, such as dyspnea, significantly decreased exercise tolerance, and/or pulmonary rales and edema; and heart failure diagnosed by a combination of physical examination, echocardiography, and chest radiography. Patients who were not admitted with heart failure but who developed the above-mentioned symptoms of heart failure during hospitalization were also included in the study. In total, 5.96% of patients were excluded because of missing data, including four patients with no data for ejection fraction and 105 with no calculation of the QRS fraction. All study participants provided written informed consent (Fig. 1).

### 2.2 Data Collection and Definitions

Data were collected for this study using a case report form specifically designed by our research group for patients with heart failure. Basic patient information was recorded, including demographic characteristics, time of admission, time of discharge, clinical diagnosis, comorbidities, underlying etiology, findings on physical examination, ECG findings, laboratory results, imaging findings (including ECG and echocardiogram), and medication. All patients were followed up by telephone every 3 months after discharge. Follow-up included supervision of medication, health education, and lifestyle guidance. Follow-up data, including mortality and time of death, were collected up to January 2020. Deaths were classified as all-cause, cardiovascular, non-cardiovascular, and unexplained.

Transthoracic echocardiography was performed by trained sonographers using a Vivid E95 ultrasound machine (GE Healthcare, Little Chalfont, United Kingdom). On most occasions, LVEF was assessed on two-dimensional images using the modified Simpson’s method of discs.

Cardiovascular death was defined as death caused by ischemic heart disease, sudden cardiac death, heart failure, or other disease affecting the cardiovascular system.

Heart failure is a clinical syndrome characterized by typical symptoms (e.g., breathlessness, ankle swelling, and fatigue) that may be accompanied by signs (e.g., elevated jugular venous pressure, pulmonary crackles, and peripheral edema) of a structural and/or functional cardiac abnormality, resulting in reduced cardiac output and/or elevated intracardiac pressures at rest or during stress.

HFpEF was defined as an ejection fraction ≥50%, an increased B-type natriuretic peptide (BNP) level (>35 pg/mL and/or NT-proBNP >125 pg/mL) associated with structural heart disease, namely, LVH and/or left atrial enlargement, or diastolic dysfunction [27]. HFrEF was defined as EF <40% with signs and symptoms of heart failure [27]. HFmrEF was defined as EF of 40%–49% with an increased BNP level (>35 pg/mL and/or NT-proBNP >125 pg/mL) and associated with structural heart disease, LVH and/or left atrial enlargement, or diastolic dysfunction [27].

### 2.3 Calculation of QRS Fraction

**Fig. 2.**
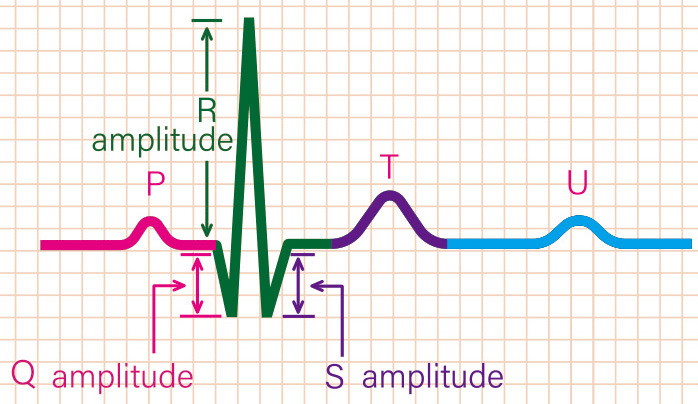
**Variables of the QRS fraction**. In this figure, the amplitude of Q wave is 3.5 mm, that of R wave is 10 mm, and that of S wave is 3.5 mm. Therefore, the total amplitude of QRS wave is 3.5 + 10 + 3.5 = 17 mm. Note: the amplitudes of Q, R and S waves are absolute, regardless of the positive and negative directions. QRS fraction = ∑R / ∑QRS × 100%.

A 12-lead surface ECG was obtained for all patients at the time of admission (standard calibration, 10 mm/1 mV; paper speed, 25 mm/s; filters, 0.05 to 0.15–100 Hz). The QRS fraction was calculated as the ratio of the total amplitude of R waves to the absolute total amplitude of QRS complexes (∑R/∑QRS) on a 12-lead ECG, i.e., QRS fraction = ∑R/∑QRS × 100%. Using the PR segment as the baseline, individual leads were analyzed by measuring the amplitude of R waves, S/s waves, and Q/q waves in all anterior chest and limb leads. ∑R was the total amplitude of R waves in each lead. ∑QRS was the absolute total amplitude of the Q/q, R, and S/s waves (Fig. 2). The QRS fractions were analyzed by two blinded cardiologists. The patients were divided into groups according to quartile of QRS fraction. Patients in the lower quartile (<43.8%), middle quartile (43.8%–61.0%), and higher quartile (>61.0%) were designated as group Q1, group Q2, and group Q3, respectively.

### 2.4 Statistical Analysis

Continuous variables are reported as the median and interquartile range. Groups were compared using a non-parametric (Kruskal-Wallis) test. Categorical variables are reported as the number (percentage). Intergroup comparisons were made using the chi-squared test. Survival curves were plotted using the Kaplan-Meier method and log-rank test to determine the significance of differences in survival according to the QRS fraction. Baseline variables that were considered clinically relevant or showed a univariate relationship with cardiovascular mortality were entered into a multivariate Cox proportional hazards regression model. Given the number of events available, the variables included were chosen carefully to ensure parsimony of the final model. Hazard ratios for cardiovascular mortality were calculated according to QRS fraction subgroup and further significant covariables with 95% confidence intervals (CIs). All statistical analyses were performed using SPSS statistical software version 24.0 (IBM Corp., Armonk, NY, USA). A *p*-value < 0.05 was considered statistically significant.

## 3. Results

### 3.1 Baseline Characteristics

Four of 1828 patients who met the study inclusion criteria were excluded for being younger than 18 years, and 109 were excluded because of missing data, leaving 1715 study participants (Fig. 1); 431 patients (25.13%) in the Q1 group, 850 (49.56%) in the Q2 group, and 434 (25.31%) in the Q3 group. In total, 1051 (61.28%) of the 1715 study participants were male. The median patient age was 66 years (56, 73); 68.75% had class III–IV heart failure, the main cause of which was coronary artery disease (54.75%). Past medical history included hypertension in 62.45% of cases. The patient background characteristics are shown in Table 1. Patients in the Q1 group were younger and contained a relatively large proportion of men. The patients in this group had a relatively low systolic blood pressure and relatively rapid heart rate, and the majority had New York Heart Association class III–IV heart failure (82.6%). The BNP level and left atrial and left ventricular inner diameters were larger in the Q1 group than in the Q2 and Q3 groups. Furthermore, chronic renal insufficiency was common in the Q1 group (detected in 59.63% of patients). The main etiology of heart failure was coronary heart disease (53.83%) followed by dilated cardiomyopathy (22.74%). There was no significant difference in the use of angiotensin-converting enzyme inhibitors (ACEI)/angiotensin receptor blockers (ARB), beta-blockers, or aldosterone receptor antagonists among the three groups. However, use of oral diuretic agents was more common in the Q1 group.

**Table 1.** **Baseline characteristics and drug treatment of patients according to QRS fraction**.

Variable		Total (n = 1715)	Q1 (n = 431)	Q2 (n = 850)	Q3 (n = 434)	*p*
Male gender [n (%)]		1051 (61.28)	293 (67.98)	529 (62.24)	229 (52.76)	0.000
SBP (mmHg)		126 (113, 142)	122 (107, 134)	126 (113, 142)	129 (116, 146)	0.000
DBP (mmHg)		78 (70, 87)	78 (68, 86)	78 (70, 87)	77 (70, 86)	0.231
HR (b.p.m.)		81 (70, 97)	83 (72, 98)	81 (70, 97)	78 (68, 94)	0.004
Hb (g/L)		133 (119, 145)	135 (123, 146)	133 (119, 145)	131 (117, 142)	0.004
WBC (×${\mathrm{10}}^{9}$/L)		7.1 (5.8, 8.9)	7 (5.82, 8.9)	7.29 (5.9, 9.2)	7.1 (5.7, 8.4)	0.026
HsCRP (mg/L)		3.8 (0.9, 12.03)	5.9 (2.31, 15.4)	5.3 (1.9, 15.8)	4.8 (1.9, 13.75)	0.153
FBG (mmol/L)		5.5 (4.73, 7.09)	5.3 (4.52, 6.7)	5.66 (4.87, 7.32)	5.39 (4.73, 6.63)	0.000
TC (mmol/L)		3.91 (3.22, 4.69)	3.79 (3.18, 4.59)	3.91 (3.22, 4.69)	4.03 (3.27, 4.77)	0.165
LDL-C (mmol/L)		2.48 (1.94, 3.09)	2.44 (1.86, 3.02)	2.47 (1.96, 3.06)	2.55 (2, 3.17)	0.198
Serum sodium (mmol/L)		140 (137, 142.3)	140 (137.1, 142.6)	139.45 (137, 142)	140.6 (138, 142.7)	0.000
Serum potassium (mmol/L)		4.04 (3.71, 4.37)	4.05 (3.67, 4.41)	4.05 (3.71, 4.36)	4.02 (3.73, 4.36)	0.956
Serum calcium (mmol/L)		2.25 (2.16, 2.34)	2.24 (2.14, 2.34)	2.24 (2.16, 2.34)	2.26 (2.15, 2.34)	0.595
Creatinine (umol/L)		81 (65, 101)	87 (72, 110)	79 (63.9, 100)	77 (63, 94.48)	0.000
AST (U/L)		25.1 (18.7, 45)	25.6 (18.7, 46.7)	26 (19, 47.45)	23 (17.88, 36.05)	0.000
ALT (U/L)		22.6 (14.8, 39.9)	23.3 (15.7, 42.8)	23.7 (15, 40.75)	19.35 (13.4, 34.03)	0.001
GGT (U/L)		31 (20, 56)	35 (23, 65)	32 (19, 58)	27 (18, 47.05)	0.000
BNP (pg/mL)		961.9 (527, 1283.64)	1080 (674, 1581.56)	956.29 (630.22, 1249.91)	841.5 (352.5, 1150)	0.000
LAD (mm)		41 (36, 46)	42 (37, 47)	41.62 (37, 45)	40 (36, 45)	0.005
LVEDD (mm)		55 (48, 64)	60 (52, 69)	54 (48, 62)	52 (46, 62)	0.000
EF (%)		45 (35, 58.82)	37.28 (30.56, 45.92)	46 (36, 58.52)	55 (40.88, 61.7)	0.000
Age (years)						0.036
	<60	541 (31.55)	154 (35.73)	272 (32)	115 (26.5)	
	60–69	536 (31.25)	134 (31.09)	264 (31.06)	138 (31.8)	
	≥70	638 (37.2)	143 (33.18)	314 (36.94)	181 (41.71)	
BMI (kg/$m^{2}$)						0.784
	<24	672 (39.25)	164 (38.14)	340 (40.05)	168 (38.8)	
	24–27.99	768 (44.86)	202 (46.98)	376 (44.29)	190 (43.88)	
	≥28	272 (15.89)	64 (14.88)	133 (15.67)	75 (17.32)	
Cardiac function classification III–IV [n (%)]		1179 (68.75)	356 (82.6)	560 (65.88)	263 (60.6)	0.000
Medical history [n (%)]						
	Coronary heart disease	943 (54.99)	236 (54.76)	487 (57.29)	220 (50.69)	0.079
	Myocardial infarction	499 (29.1)	126 (29.23)	270 (31.76)	103 (23.73)	0.011
	Hypertension	1071 (62.45)	255 (59.16)	524 (61.65)	292 (67.28)	0.038
	Diabetes	588 (34.29)	134 (31.09)	321 (37.76)	133 (30.65)	0.011
	Stroke and TIA	261 (15.22)	56 (12.99)	132 (15.53)	73 (16.82)	0.275
	Chronic renal insufficiency with intervention	860 (50.15)	257 (59.63)	411 (48.35)	192 (44.24)	0.000
Etiological diagnosis of heart failure [n (%)]						
	Coronary disease	939 (54.75)	232 (53.83)	491 (57.76)	216 (49.77)	0.022
	Hypertension	191 (11.14)	60 (13.92)	77 (9.06)	54 (12.44)	0.020
	Valvular disease	272 (15.86)	60 (13.92)	140 (16.47)	72 (16.59)	0.444
	Dilated cardiomyopathy	265 (15.45)	98 (22.74)	117 (13.76)	50 (11.52)	0.000
	Hypertrophic cardiomyopathy	20 (1.17)	3 (0.7)	10 (1.18)	7 (1.61)	0.454
	Pericardial disease	5 (0.29)	1 (0.23)	2 (0.24)	2 (0.46)	0.751
	other	369 (21.52)	79 (18.33)	181 (21.29)	109 (25.12)	0.051
Drug treatment [n (%)]						
	Diuretics, oral	1606 (93.64)	423 (98.14)	789 (92.82)	394 (90.78)	0.000
	ACE inibitors/ARBs	986 (57.49)	241 (55.92)	500 (58.82)	245 (56.45)	0.536
	Beta-blockers	1311 (76.44)	322 (74.71)	663 (78)	326 (75.12)	0.319
	Aldosterone receptor antagonist	1451 (84.61)	373 (86.54)	721 (84.82)	357 (82.26)	0.211

ACEI, angiotensin-converting enzyme inhibitors; ALT, alanine aminotransferase; ARB, angiotensin receptor blockers; AST, aspartate aminotransferase; BNP, brain natriuretic peptide; CV, cardiovascular; DBP, diastolic pressure; FBS, fasting blood glucose; GGT, glutamyl transferase; Hb, hemoglobin; HR, heart rate; HsCRP, high-sensitivity C-reactive protein; LAD, left atrial diameter; LDL-C, low-density lipoprotein cholesterol; LVEDD, left ventricular end-diastolic diameter; LVEF, left ventricular ejection fraction; SBP, systolic pressure; TC, total cholesterol; TIA, transient ischemic attack; WBC, white blood cells. 1 mmHg = 0.133 kPa.

### 3.2 Findings during Follow-Up

The median duration of follow-up in the 1715 patients (93.8% of the study cohort) was 261 (39, 502) days. In total, there were 341 deaths (19.88%), which included 282 (16.44%) cardiovascular, 33 (1.92%) non-cardiovascular, and 26 (1.52%) unexplained deaths. An inverse relationship between cardiovascular mortality and QRS fraction subgroup was observed: mortality was highest in group Q1 (20.42%) followed by group Q2 (15.41%) and was lowest in group Q3 (14.52%) (*p *< 0.05; Table 2).

**Table 2.** **Outcomes according to QRS fraction**.

	Total (n = 1715)	Q1 (n = 431)	Q2 (n = 850)	Q3 (n = 434)	*p*
All causes of death	341 (19.88)	95 (22.04)	161 (18.94)	85 (19.59)	0.415
CV death	282 (16.44)	88 (20.42)	131 (15.41)	63 (14.52)	0.034
Non-CV death	33 (1.92)	3 (0.7)	16 (1.88)	14 (3.23)	0.025
Unknown	26 (1.52)	4 (0.93)	14 (1.65)	8 (1.84)	0.495

CV, cardiovascular; Q, QRS fraction subgroup.

**Fig. 3.**
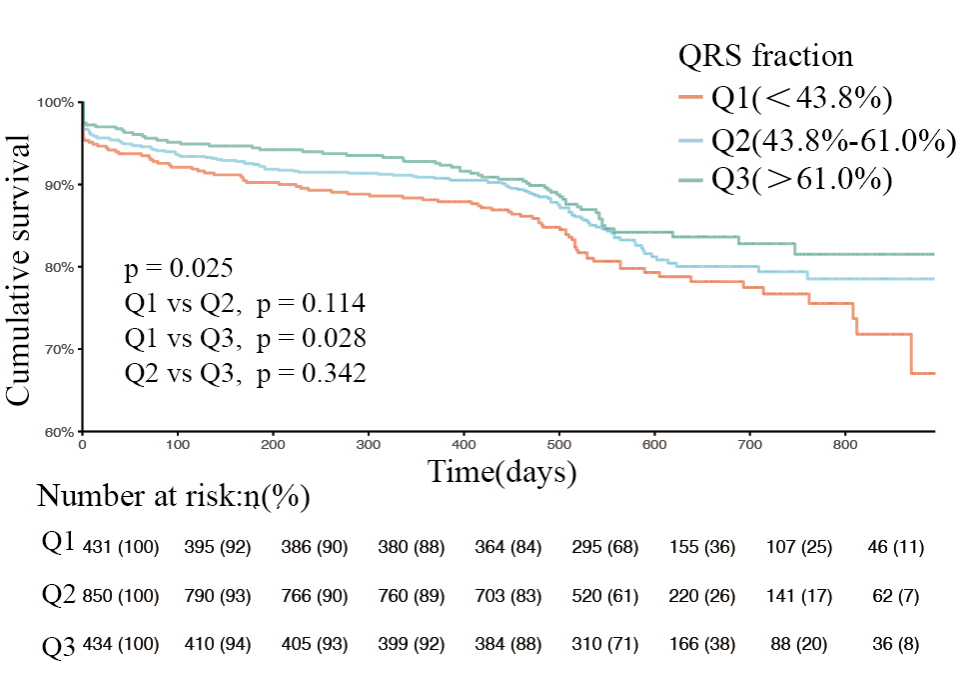
**Kaplan-Meier curves of patients with heart failure at different QRS fraction levels (log-rank test *p* = 0.025)**. Q1, QRS fraction <43.8%. Q2, QRS fraction (43.8%–61.0%). Q3, QRS fraction >61.0%. *p* = 0.114, Q1 compared with Q2. *p* = 0.028, Q1 compared with Q3. *p* = 0.342, Q2 compared with Q3.

Variables that were considered clinically meaningful (sex, age, body mass index, etiology of coronary disease, myocardial infarction, stroke and transient ischemic attack, hypertension, chronic renal insufficiency, hemoglobin, aspartate aminotransferase, alanine aminotransferase, total cholesterol, low-density lipoprotein cholesterol, fasting blood glucose, BNP, serum sodium, left atrial diameter, left ventricular end-diastolic diameter, and use of diuretics and ACEIs/ARBs) (Table 3) were examined in univariate analysis and subsequently in a multivariate Cox regression model to identify independent predictors of cardiovascular causes of mortality. Multiple variables, including age, myocardial infarction, hemoglobin, serum sodium, left atrial diameter, BNP on admission, QRS fraction, and use of ACEIs/ARBs were significantly and independently associated with cardiovascular mortality (Table 4). The risk of cardiovascular death was lower in the Q2 and Q3 groups than in the Q1 group, with hazard ratios of 0.668 (95% CI 0.457–0.974, *p* = 0.036) and 0.538 (95% CI 0.341–0.849, *p* = 0.008), respectively (Table 4). Kaplan-Meier survival curves of patients with heart failure at different QRS levels was shown in Fig. 3 (*p* = 0.025, log-rank test).

**Table 3.** **Predictors of cardiovascular mortality in univariable analysis**.

Variable		HR (95% CI)	*p*
${\mathrm{Age (years)}}^{\text{a}}$			
	60–69	1.65 (1.149–2.368)	0.007
	≥70	3.569 (2.586–4.926)	0.000
${\mathrm{QRS (\%)}}^{\text{b}}$			
	Q2	0.803 (0.612–1.052)	0.111
	Q3	0.695 (0.503–0.961)	0.028
BMI (kg/$m^{2}$)^c^			
	24–27.9	0.734 (0.575–0.936)	0.013
	≥28	0.364 (0.232–0.571)	0.000
Hb (g/L)		0.983 (0.978–0.988)	0.000
FBG (mmol/L)		1.006 (1.003–1.010)	0.000
TC (mmol/L)		0.880 (0.788–0.982)	0.022
LDL-C (mmol/L)		0.776 (0.670–0.899)	0.001
Serum Sodium (mmol/L)		0.981 (0.974–0.988)	0.000
AST (U/L)		1.000 (1.000–1.001)	0.004
ALT (U/L)		1.000 (1.000–1.001)	0.022
Coronary heart disease		1.296 (1.022–1.644)	0.033
Hypertension		0.771 (0.609–0.975)	0.030
Chronic renal insufficiency with intervention		1.802 (1.414–2.297)	0.000
Myocardial infarction		1.495 (1.172–1.908)	0.001
Stroke and TIA		1.646 (1.237–2.189)	0.001
BNP (pg/mL)		1.000 (1.000–1.000)	0.000
LAD (mm)		1.021 (1.009–1.033)	0.001
LVEDD (mm)		1.003 (1.001–1.005)	0.007
Diuretics, oral		3.741 (1.545–9.06)	0.003
ACE inhibitors/ARBs		0.611 (0.483–0.772)	0.000
Cardiac function classification III–IV		2.528 (1.832–3.487)	0.000
LVEF (%)		0.987 (0.978–0.995)	0.003

^a^Reference value age <60 years. ^b^Reference value QRS <43.8%. ^c^Reference value BMI <24 kg/$m^{2}$. ACEI, angiotensin-converting enzyme inhibitors; ALT, alanine aminotransferase; ARB, angiotensin receptor blockers; AST, aspartate aminotransferase; BMI, body mass index; FBG, fasting blood glucose; Hb, hemoglobin; LAD, left atrial diameter; LDL-C, low-density lipoprotein cholesterol; LVEDD, left ventricular end-diastolic diameter; LVEF, left ventricular ejection fraction; TC, total cholesterol. 1 mmHg = 0.133 kPa.

**Table 4.** **Predictors of cardiovascular mortality in multivariable analysis**.

Variable		HR (95% CI)	*p*
${\mathrm{QRS (\%)}}^{\text{a}}$			
	Q2	0.668 (0.457–0.974)	0.036
	Q3	0.538 (0.341–0.849)	0.008
${\mathrm{Age (years)}}^{\text{b}}$			
	60–69	1.315 (0.800–2.161)	0.280
	≥70	2.514 (1.594–3.966)	0.000
Myocardial infarction		1.457 (1.018–2.086)	0.040
ACE inhibitors/ARBs		0.670 (0.477–0.942)	0.021
Hypertension		0.713 (0.503–1.009)	0.056
Hb (g/L)		0.983 (0.976–0.990)	0.000
Serum Sodium (mmol/L)		0.985 (0.972–0.997)	0.017
BNP (pg/mL)		1.000 (1.000–1.000)	0.000
LAD (mm)		1.026 (1.012–1.041)	0.000

^a^Reference value QRS <43.8%. ^b^Reference value age <60 years. ACEI, angiotensin-converting enzyme inhibitors; ARB, angiotensin receptor blockers; BNP, brain natriuretic peptide; Hb, hemoglobin; LAD, left atrial diameter.

**Fig. 4.**
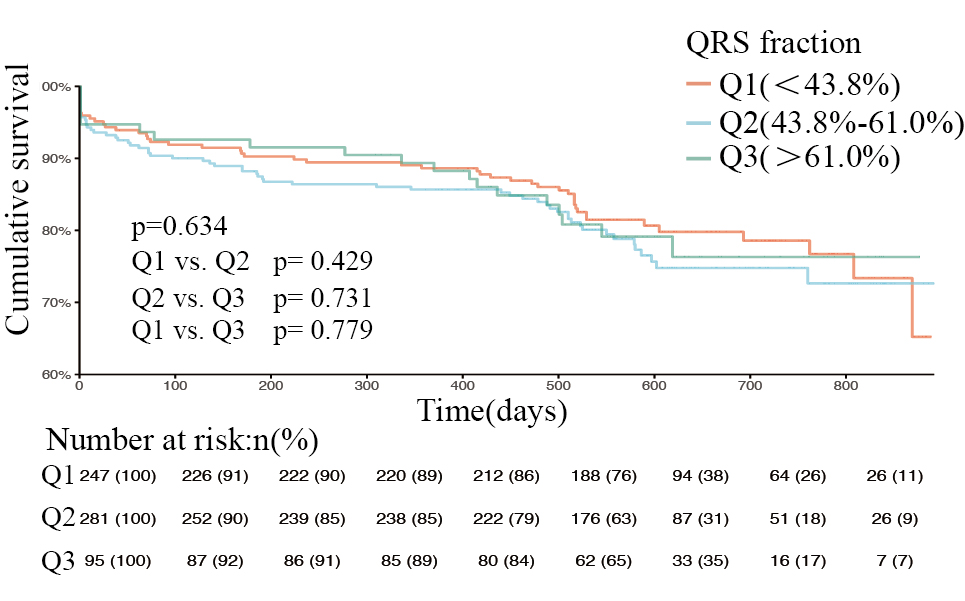
**Kaplan-Meier curves of patients with reduced ejection fraction at different QRS levels (log-rank test *p* = 0.634)**. Q1, QRS fraction <43.8%. Q2, QRS fraction (43.8%–61.0%). Q3, QRS fraction >61.0%. *p* = 0.429, Q1 compared with Q2. *p* = 0.731, Q2 compared with Q3. *p* = 0.779, Q1 compared with Q3.

**Fig. 5.**
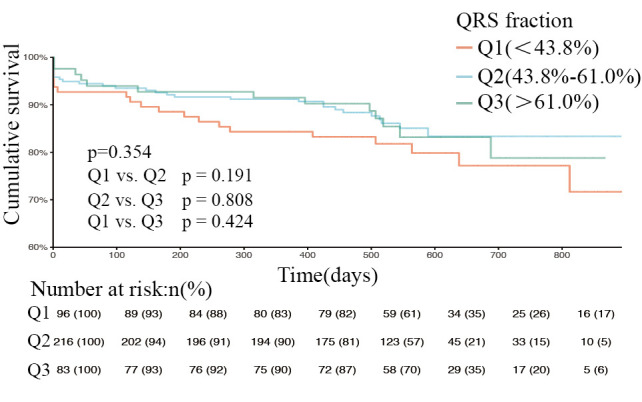
**Kaplan-Meier curves of patients with mid-range ejection fraction at different QRS levels (log-rank test *p* = 0.354)**. Q1, QRS fraction <43.8%. Q2, QRS fraction (43.8%–61.0%). Q3, QRS fraction >61.0%. *p* = 0.191, Q1 compared with Q2. *p* = 0.808, Q2 compared with Q3. *p* = 0.424, Q1 compared with Q3.

**Fig. 6.**
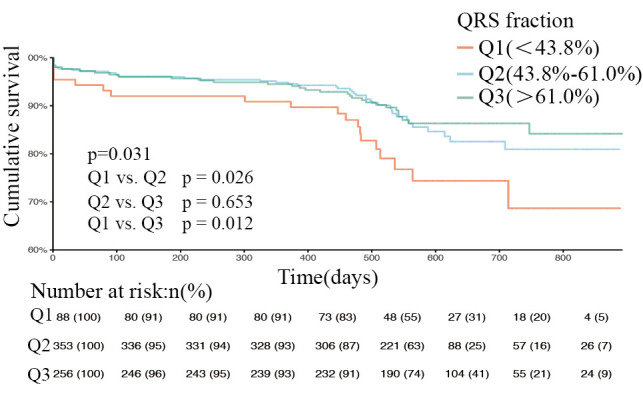
**Kaplan-Meier curves of patients with preserved ejection fraction at different QRS levels (log-rank test *p* = 0.031)**. Q1, QRS fraction <43.8%. Q2, QRS fraction (43.8%–61.0%). Q3, QRS fraction >61.0%. *p* = 0.026, Q1 compared with Q2. *p* = 0.653, Q2 compared with Q3. *p* = 0.012, Q1 compared with Q3.

The overall study population was then stratified according to type of heart failure, and the respective proportions of HFrEF, HFmEF, and HFpEF were 697 (40.6%), 395 (23%), and 623 (36.3%); Figs. 4,5,6 show the respective survival curves for these three groups (*p* = 0.634, 0.354, and 0.031, log-rank test). In the HFpEF subgroup, the cumulative survival rate was significantly higher in the Q2 and Q3 groups than in the Q1 group, with no significant difference in survival between the Q2 and Q3 groups. However, in the HFrEF and HFmEF groups, there was no significant difference in cumulative survival according to the QRS fraction (*p *> 0.05). **Supplementary Table 1** shows the factors that were independently associated with cardiovascular mortality in the the three HF groups. Besides, we compared the survival rate in the three HF groups according to the quartile of QRS fraction (*p* = 0.483, 0.003, and 0.045, log-rank test; **Supplementary Figs. 1–3**).

## 4. Discussion

A significant correlation between the sum of the R wave amplitudes on the orthogonal ECG and LVEF, meaning that the ejection fraction could be estimated on the ECG, was first reported by Gottwlk *et al*. [29] in 1978. However, in 1983, Luweart *et al*. [30] advised against use of the ∑R or ∑QRS alone to estimate ejection fraction because these values could be influenced by many factors, such as left intraventricular pressure, left ventricular wall thickness, and left ventricular size. Our group has previously demonstrated that the ∑R/∑QRS, or QRS fraction, is a better method for assessment of ejection fraction [25, 26] and that it is not affected by the presence of myocardial infarction or myocardial hypertrophy and reduces or eliminates the effect of individual patient factors, such as chest wall thickness and skin conductivity. This study is the first to investigate the ability of the QRS fraction on a 12-lead ECG to predict the long-term prognosis in patients with heart failure. Its findings show that hyponatremia, elevated BNP/NT-proBNP levels, anemia, and an increased left atrial internal diameter are independent risk factors for a poor prognosis in patients with heart failure and are consistent with the results of previous studies [31, 32, 33, 34, 35]. We also found that use of an ACEI/ARB was associated with a reduced risk of cardiovascular death. Overall, the QRS fraction was still independently associated with the risk of cardiovascular death after adjustment for the above-mentioned clinically meaningful covariates known to predict the prognosis. The QRS fraction can also help to identify patients with heart failure who are at high risk of cardiovascular death and allow targeted interventions for controllable risk factors, such as blood pressure, serum sodium, and hemoglobin, which may help to improve the prognosis.

In recent years, significant advances have been made in the diagnosis and treatment of heart failure, and the management of patients with this condition has become increasingly sophisticated. However, 70% of patients with heart failure are aged >65 years with preserved ejection fraction [36, 37, 38], and how to improve the outcomes in these patients is an urgent problem to be solved. 12-lead ECG markers have been used to risk-stratify patients with HFpEF in an effort to improve their prognosis. The ECG Cornell product (CP) is defined as CP = ([RaVL + SV3] × QRS duration) and is calculated from the R wave amplitude in aVL (RaVL), the S wave depth in V3 (SV3), and the QRS duration. Previous studies have shown that the CP can predict the prognosis in patients with CHF [39] and those with HFpEF [40]. However, the CP is associated with LVH and influenced by body size and heart size. In contrast, the QRS fraction is not affected by LVH when predicting cardiovascular mortality in patients with HFpEF. The QRS scoring system estimates fibrotic scarring by measuring changes in Q-wave, R-wave, and S-wave durations, amplitudes, and morphologies [41, 42] and is a strong predictor of cardiovascular mortality [43]. Strauss *et al*. [44] proposed using the QRS scoring method and analysis of the QRS-T angle to identify patients with predominantly preserved LVEF and a high risk of 1-year mortality. However, the QRS scoring method is cumbersome and difficult to use in clinical practice and the QRS-T angle is affected by bundle branch block. In this study, we found that the QRS fraction was simple to calculate and there were no changes in our results when patients with bundle branch block were excluded (**Supplementary Figs. 4–7**). Therefore, the QRS fraction may have applications in a wider patient population. However, given that the proportion of patients with bundle branch block in this study was small, we plan to investigate whether or not the QRS fraction is affected by bundle branch block in more detail in the future.

The role of the QRS fraction in patients with HFpEF is not well understood. On the one hand, the QRS fraction mainly reflects the amplitude of each component of the QRS wave and is the ratio of a comprehensive 12-lead vector. Therefore, we consider that the QRS fraction has an advantage over other ECG markers of heart failure. Moreover, it is not population-specific. On the other hand, it is clear that the mechanisms underlying HFpEF are related to intermittent pressure overload, coronary microvascular dysfunction, tissue ischemia, and fibrosis [37, 45], which may affect findings on a routine ECG and be closely related to the QRS fraction.

ECG markers are now recognized as important predictors of the prognosis in patients with heart failure. After more extensive and in-depth validation in clinical trials, the QRS fraction should become an easily accessible clinical measurement on the 12-lead ECG and make it possible to identify patients with high-risk HFpEF. The QRS fraction is a simple, convenient, and practical index with the potential to be useful in clinical practice and will hopefully be used widely.

## 5. Conclusions

Compared with biomarkers and echocardiography, the QRS fraction is a simple and non-invasive parameter that may serve as a prognostic indicator of the long-term risk of cardiovascular death in patients with heart failure, especially those with HFpEF.

## 6. Limitations

This study had some limitations. First, it had a prospective cohort design and was conducted at a single center. Multicenter studies are needed to increase the credibility of our present findings by expanding the sample size. Second, the mean follow-up duration was short and should be extended in future studies of the long-term prognosis in patients with heart failure. Finally, we did not record continuous changes in the QRS fraction, and the next step is to include ECG examinations during follow-up of patients to observe the relationship between continuous changes in the QRS fraction and the prognosis.

## Supplementary Material

Supplementary material associated with this article can be found, in the online version, at https://doi.org/10.31083/j.rcm2307241.
