# Supplementary material for: Predictive Value of QRS Fraction for Cardiovascular Death in Patients with Heart Failure: A Prospective Cohort Study in Acute Decompensated Heart Failure (Heb-ADHF)
Source: Rev Cardiovasc Med. 2022 Jun 27;23(7):241. doi: 10.31083/j.rcm2307241 (PMC11266833; doi:10.31083/j.rcm2307241)
Supplement: Supplementary file 1 [file 2153-8174-23-7-241-s1.pdf]

Supplement 1 Multivariable predictors for CV death mortality by ejection fraction category

Variable	HFrEF		HFmrEF		HFpEF	
	HR (95% CI)	*P*	HR (95% CI)	*P*	HR (95% CI)	*P*
QRS(%)[a]						
Q2	–	–	–	–	0.397(0.21-0.749)	0.004
Q3	–	–	–	–	0.371(0.19-0.724)	0.004
Age (years)[b]						
60-69	0.787(0.389-1.591)	0.504	–	–	–	–
⩾70	1.98(1.058-3.706)	0.033	–	–	–	–
BMI (kg/m^2)[c]						
24-27.9	–	–	–	–	0.762(0.453-1.282)	0.306
⩾28	–	–	–	–	0.172(0.041-0.722)	0.016
Myocardial infarction	–	–	3.731(1.304-10.675)	0.014	1.947(1.062-3.571)	0.031
Hb（g/L）	–	–	0.969(0.944-0.996)	0.022	0.982(0.972-0.992)	0.000
ACE inibitors/ARBs	0.373(0.223-0.624)	0.000	–	–	–	–
BNP (pg/mL)	1(1-1)	0.003	1(1-1.001)	0.050	1(1-1)	0.010
Sodium (mmol/L)	0.904(0.866-0.944)	0.000	–	–	–	–
LAD (mm)	–	–	1.089(1.03-1.152)	0.003	1.029(1.013-1.046)	0.000
Stroke and TIA	1.876(1.015-3.469)	0.045	–	–	–	–
Creatinine（umol/L）	–	–	1.007(0.999-1.015)	0.067	–	–

Adjused for gender,age,coronary disease,SBP,DBP,Hb,WBC,FBP,AST,ALT,BNP,TC,LDL-c,Sodium,Creatinine,BUN,BNP,BMI,LAD,LVEDD,QRS fraction,myocardial infarction,stroke and TIA,Diuretics,ACE inibitors/ARBs.

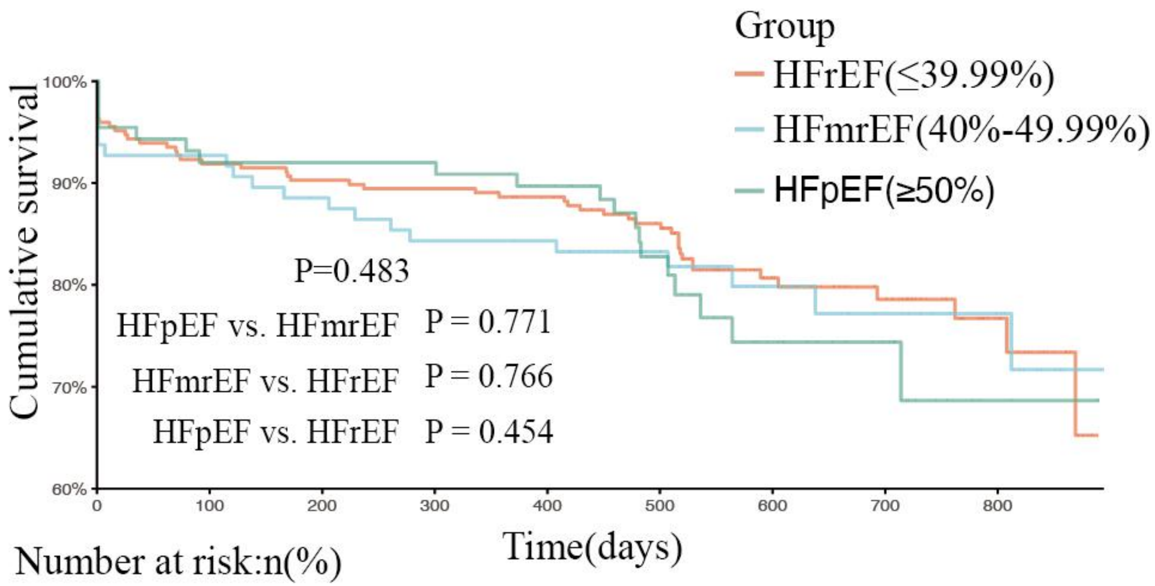

Number at risk:n(%)

HFrEF	247 (100)	226 (91)	222 (90)	220 (89)	212 (86)	188 (76)	94 (38)	64 (26)	26 (11)
HFmrEF	96 (100)	89 (93)	84 (88)	80 (83)	79 (82)	59 (61)	34 (35)	25 (26)	16 (17)
HFpEF	88 (100)	80 (91)	80 (91)	80 (91)	73 (83)	48 (55)	27 (31)	18 (20)	4 (5)

Supplementary Fig 1. KM plots of patients with lower QRS fraction at different ejection fraction levels.

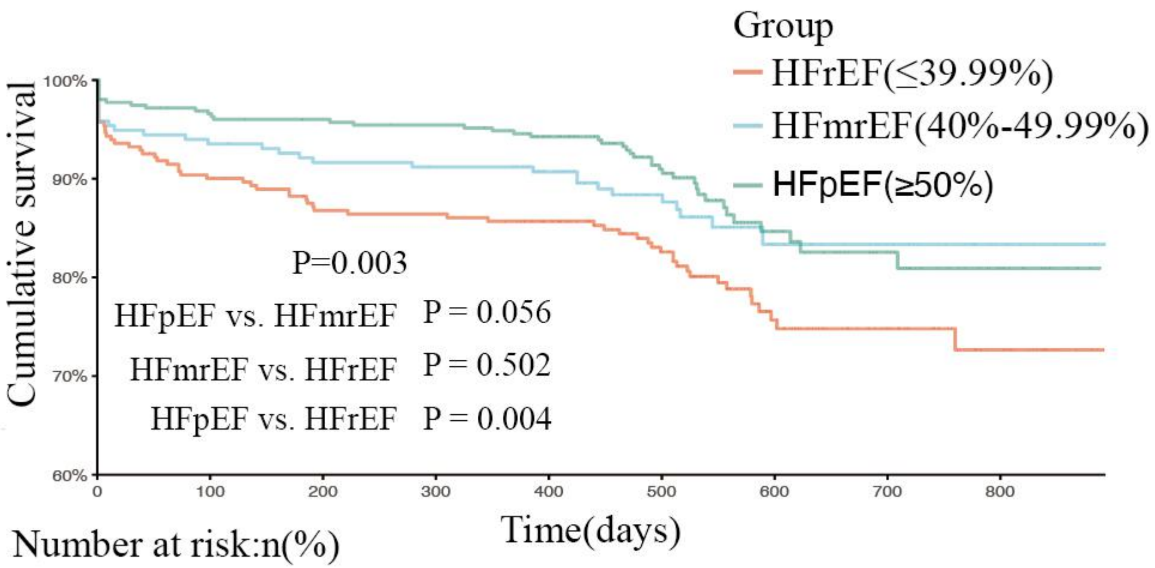

Number at risk:n(%)

HFrEF	281 (100)	252 (90)	239 (85)	238 (85)	222 (79)	176 (63)	87 (31)	51 (18)	26 (9)
HFmrEF	216 (100)	202 (94)	196 (91)	194 (90)	175 (81)	123 (57)	45 (21)	33 (15)	10 (5)
HFpEF	353 (100)	336 (95)	331 (94)	328 (93)	306 (87)	221 (63)	88 (25)	57 (16)	26 (7)

Supplementary Fig 2. KM plots of patients with middle QRS fraction at different ejection fraction levels.

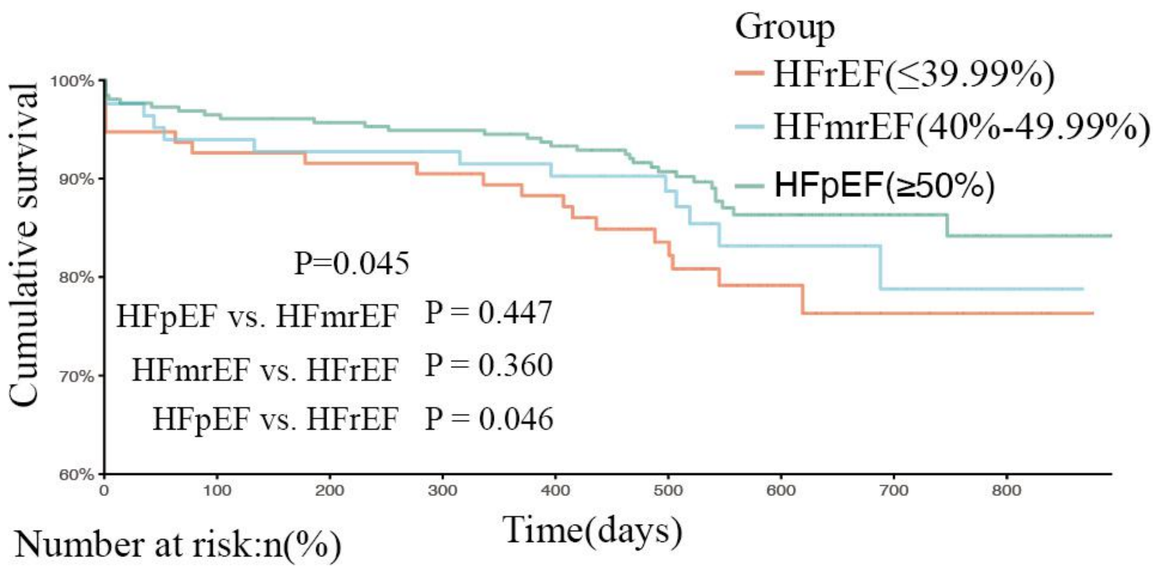

Number at risk:n(%)

HFrEF	95 (100)	87 (92)	86 (91)	85 (89)	80 (84)	62 (65)	33 (35)	16 (17)	7 (7)
HFmrEF	83 (100)	77 (93)	76 (92)	75 (90)	72 (87)	58 (70)	29 (35)	17 (20)	5 (6)
HFpEF	256 (100)	246 (96)	243 (95)	239 (93)	232 (91)	190 (74)	104 (41)	55 (21)	24 (9)

Supplementary Fig 3. KM plots of patients with higer QRS fraction at different ejection fraction levels.

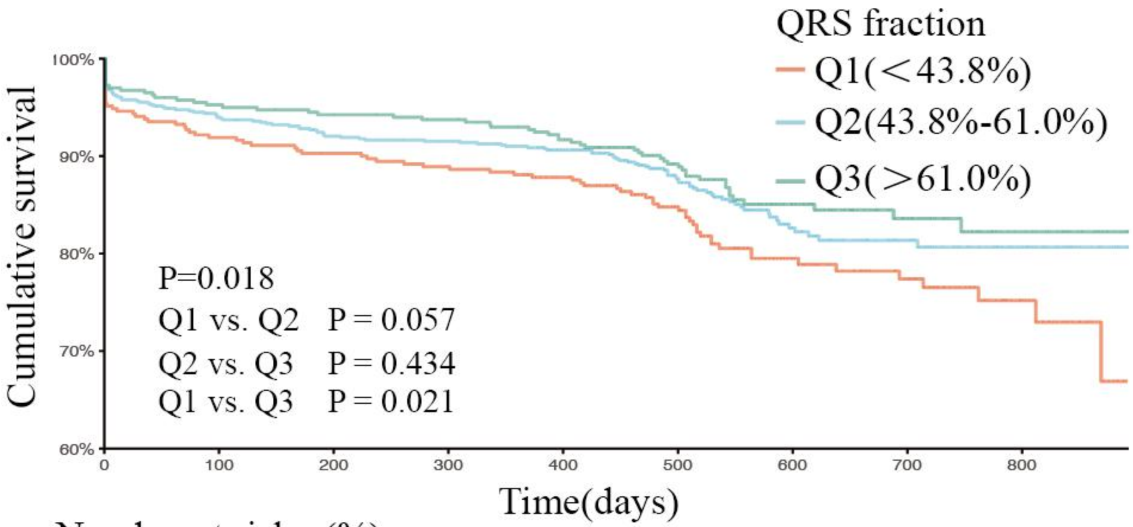

Number at risk:n(%)

Q1	371 (100)	339 (91)	332 (89)	327 (88)	311 (84)	246 (66)	131 (35)	96 (26)	39 (11)
Q2	783 (100)	730 (93)	707 (90)	701 (90)	653 (83)	478 (61)	206 (26)	130 (17)	57 (7)
Q3	401 (100)	379 (95)	374 (93)	369 (92)	354 (88)	293 (73)	160 (40)	84 (21)	34 (8)

Supplementary Fig 4. KM plots of patients with heart failure at different QRS levels

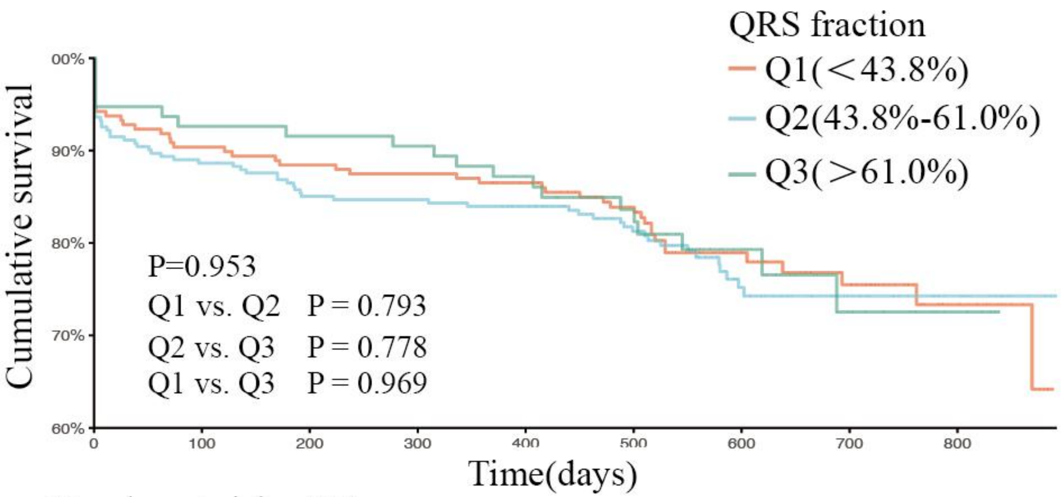

Number at risk:n(%)

Q1	208 (100)	187 (90)	183 (88)	181 (87)	173 (83)	151 (73)	80 (38)	58 (28)	22 (11)
Q2	282 (100)	249 (88)	235 (83)	234 (83)	219 (78)	170 (60)	82 (29)	47 (17)	23 (8)
Q3	95 (100)	87 (92)	86 (91)	85 (89)	78 (82)	63 (66)	34 (36)	15 (16)	5 (5)

Supplementary Fig 5. KM plots of patients with reduced ejection fraction at different QRS levels

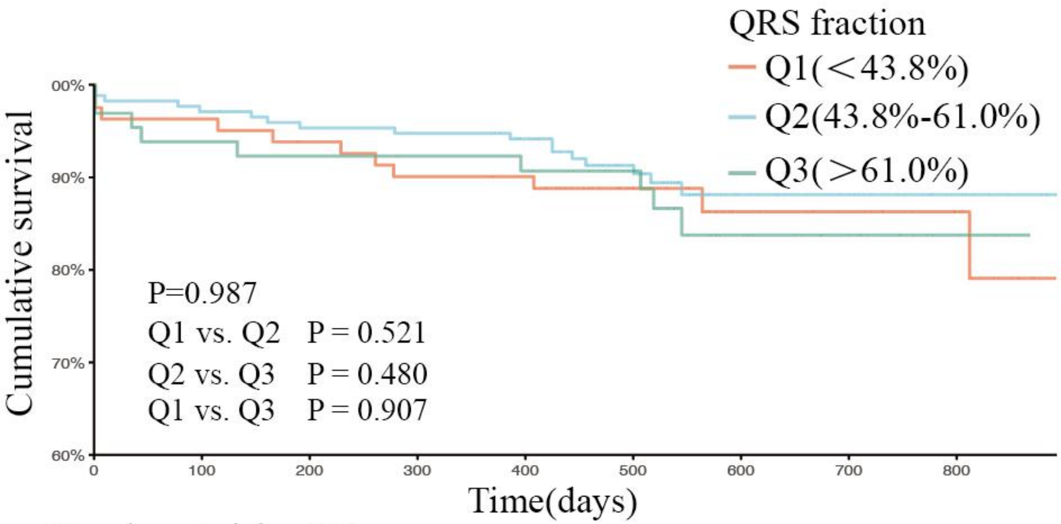

Number at risk:n(%)

Q1	81 (100)	78 (96)	75 (93)	72 (89)	71 (88)	51 (63)	27 (33)	22 (27)	14 (17)
Q2	172 (100)	167 (97)	163 (95)	161 (94)	147 (85)	101 (59)	39 (23)	28 (16)	9 (5)
Q3	65 (100)	60 (92)	59 (91)	58 (89)	57 (88)	49 (75)	23 (35)	15 (23)	5 (8)

Supplementary Fig 6. KM plots of patients with mild reduced ejection fraction at different QRS levels

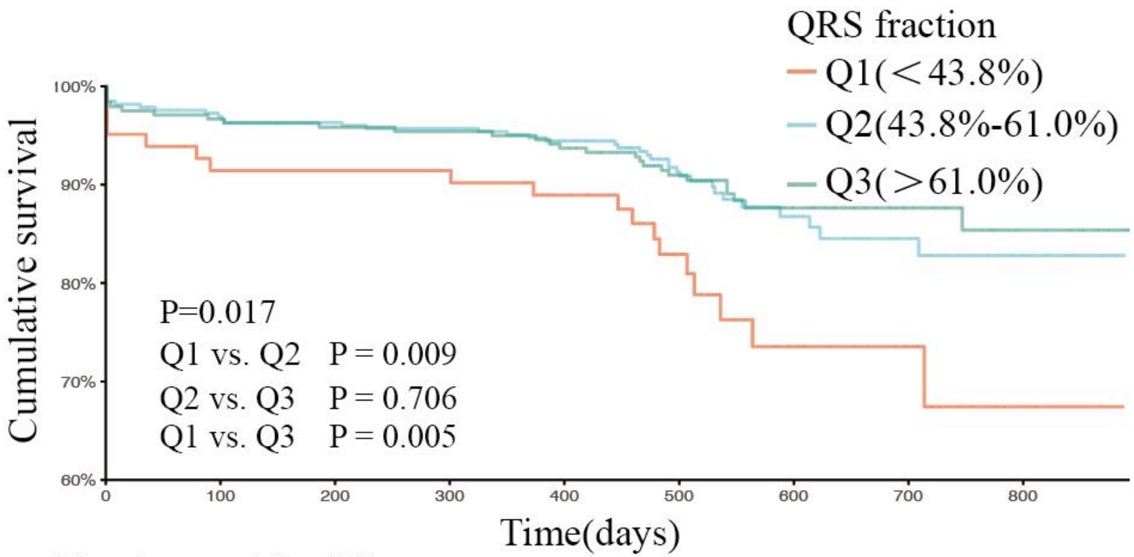

Number at risk:n(%)

Q1	82 (100)	74 (90)	74 (90)	74 (90)	67 (82)	44 (54)	24 (29)	16 (20)	3 (4)
Q2	329 (100)	314 (95)	309 (94)	306 (93)	287 (87)	207 (63)	85 (26)	55 (17)	25 (8)
Q3	241 (100)	232 (96)	229 (95)	226 (94)	219 (91)	181 (75)	103 (43)	54 (22)	24 (10)

Supplementary Fig 7. KM plots of patients with preserved ejection fraction at different QRS levels
